# A hybrid, effectiveness-implementation research study protocol targeting antenatal care providers to provide female genital mutilation prevention and care services in Guinea, Kenya and Somalia

**DOI:** 10.1186/s12913-021-06097-w

**Published:** 2021-02-01

**Authors:** Wisal Ahmed, Vernon Mochache, Karin Stein, Patrick Ndavi, Tammary Esho, Mamadou Dioulde Balde, Anne-Marie Soumah, Ahmed Diriye, Muna Abdi Ahmed, Max Petzold, Christina Pallitto

**Affiliations:** 1 Department of Sexual and Reproductive Health, World Health Organization, 20 Avenue Appia, 1211 Geneva, Switzerland; 2 Department of Obstetrics and Gynecology, University of Nairobi, Nairobi, Kenya; 3 Department of Health System Management and Public Health, Technical University of Kenya, Nairobi, Kenya; 4 Centre for Research in Reproductive Health in Guinea, Conakry, Guinea; 5 Data and Research Solutions, Hargeisa, Somalia; 6 School of Public Health and Community Medicine, Institute of Medicine, University of Gothenburg, Gothenburg, Sweden

**Keywords:** Female genital mutilation, Prevention, Health-sector involvement, Person-Centred communication, Antenatal care providers, Guinea, Kenya, Somalia

## Abstract

**Background:**

In settings with high prevalence of female genital mutilation (FGM), the health sector could play a bigger role in its prevention and care of women and girls who have undergone this harmful practice. However, ministries of health lack clear policies, strategic plans or dedicated funding to implement anti-FGM interventions. Along with limited relevant knowledge and skills to prevent the practice of FGM and care for girls and women living with FGM, health providers have limited interpersonal communication skills and self-efficacy, while some may have supportive attitudes towards FGM and its medicalization. We propose to test the effectiveness of a health system strengthening intervention that includes training antenatal care (ANC) providers on person-centred communication (PCC) for FGM prevention.

**Methods:**

This will be a two-level, hybrid, effectiveness-implementation research study using a cluster randomized trial design in Guinea, Kenya and Somalia conducted over a 6 months period. In each country, within pre-selected regions/counties, 60 ANC clinics will be randomized to intervention and control arms. At baseline, all clinics will receive the level one intervention involving provision of FGM-related clinical guidelines and handbook as well as anti-FGM policies and posters. At month 3, intervention clinics will receive the level two intervention comprising of a training for ANC providers on PCC to challenge their FGM-related attitudes and build their communication skills to effectively provide FGM prevention counselling. A process evaluation will be conducted to understand ‘how’ and ‘why’ the intervention package achieves intended results. Multi-level regression modelling will be used for quantitative data analysis while qualitative data will be assessed using thematic content analysis to determine the effectiveness, feasibility and acceptability of the different intervention levels.

**Discussion:**

The proposed study will strengthen the knowledge base regarding how to effectively involve health providers in FGM prevention and care.

**Trial registration:**

Trial registration and date: PACTR201906696419769 (June 3rd, 2019).

**Supplementary Information:**

The online version contains supplementary material available at 10.1186/s12913-021-06097-w.

## Background

Female genital mutilation (FGM) is a harmful practice affecting more than 200 million women and girls aged 15–49 years globally [1, 2]. It involves the partial or total removal of external female genitalia or other injury to female genitals for non-medical reasons. FGM has no health benefits and interferes with the body’s natural functions [3]. Despite the elevated risk of health complications among those affected by this harmful practice, in settings with high FGM prevalence, the health workforce is neither actively engaged in prevention nor well trained to provide quality care for its complications. In addition, the absence of health sector policies and strategic plans or specified funding, presents a weak environment to implement prevention and care interventions [4, 5].

If countries are to achieve the Sustainable Development Goal (SDG) 5.3 to eliminate FGM by 2030, concerted efforts by all sectors are needed [6, 7]. Health providers not only play a key role in providing quality health care to women and girls suffering the negative consequences of FGM, but as highly respected members of society, they could also play the important role of being effective change agents contributing towards the abandonment of the practice. Nevertheless, there is limited evidence to guide the health sector in implementing effective prevention and care interventions at scale [7, 8]. The few studies conducted are considered to be of low quality and not informed by any behavior change theories.

Further, health providers in some of these settings have demonstrated attitudes supportive of FGM, promoting either its medicalization or continuation of some types considered to be ‘less harmful’ [5]. A formative study conducted by the World Health Organization (WHO) and partners in three regions of Guinea (Faranah, Labé and Conakry) to understand the health system response to FGM and its medicalization, revealed low coverage of training as well as poor knowledge, self-efficacy and interpersonal communication skills among Guinean health providers.

The formative research also highlighted the demand for additional training by health care providers as well as the potential role they could play in providing effective FGM prevention counselling. These findings informed the development of the intervention package and implementation strategy to be tested in this proposed study.

Several other activities have prompted and informed the development of the proposed study; the ongoing support by WHO for the development of national, FGM-related, health sector action plans highlighted the absence of evidence based effective health sector interventions to implement at large scale as well as the lack of a clear approach or methodology to provide FGM prevention or care within routine health services. Furthermore, there are limited training tools or aides to guide health care providers to provide prevention services; country-specific situational analyses on FGM epidemiology, national FGM abandonment strategies/interventions, current legislative and political positions as well as the status of health systems; review of effective health system interventions to address FGM within the health sector; review of existing interpersonal communication training materials and consultations with communication and behaviour change experts. Finally, we conducted a pilot exercise for the training package intervention with positive feedback from midwives reporting that it challenged their beliefs on what they considered a norm and felt empowered as change agents against this practice.

The proposed study will use a rigorous design to generate evidence on the effectiveness of a health systems strengthening approach to effectively address FGM. It is planned to be implemented on the low-level health care facilities that offer primary health care services within the three proposed countries. The proposed complex intervention package includes a novel training for ANC providers (nurses/midwives) that has been developed using existing behavior change theories. This training is aimed at improving their interpersonal communication skills and addressing their FGM-related attitudes and values [9, 10]. Though there is no evidence found on unintended harms resulting from health systems interventions, we will however remain vigilant in identifying, assessing and responding to any event as per study’s standard operating procedures. In light of the recent COVID-19 pandemic, we will implement safety measures during training and data collection activities in alignment with each country’s national guidance.

The proposed study will also involve a process evaluation component to explore ‘how’ and ‘why’ the intervention achieves its intended outcomes. Should this intervention prove to be effective, it has the potential to inform implementation of health sector strategies that will contribute meaningfully to achievement of the SDG 5.3 target.

## Study objective and specific AIMS

The objective of this implementation research study is to test the effectiveness of a two-level, complex intervention package in ANC clinics at the primary health care level in three sub-Saharan African countries namely, Guinea, Kenya and Somalia.

Specifically, the study aims to achieve the following:

Primary aims:

- To determine if a health system strengthening (HSS) intervention within the ANC context improves preparedness to provide FGM prevention and care services
- To determine if training ANC providers on person-centered communication (PCC) improves delivery of FGM prevention counseling for ANC clients in primary care settings

Secondary aims:

- To determine if ANC providers who receive training on PCC for FGM prevention are more likely to have: 1) improved knowledge about FGM; 2) improved interpersonal communication skills; 3) improved self-efficacy and 4) less supportive attitudes towards FGM and its medicalization
- To understand ‘how’ and ‘why’ an intervention that uses a social norm change approach achieves its implementation outcomes, by measuring parameters such as fidelity, coverage, feasibility and acceptability of the delivered intervention

## Methods

The roles and responsibilities of all individuals and institutions involved in the study such as study staff, study investigators, steering committee, and the Data Safety and Monitoring Board (DSMB) are clearly described in the standard operating procedures.

### Study design

The proposed study is a hybrid, type-2, effectiveness-implementation research using a cluster randomized trial design [11]. The intervention being tested will involve a novel training for ANC providers to enable them to deliver person centered communication on FGM prevention while the health systems strengthening approach being evaluated includes making available health policies/directives, guidelines, posters and clinical tools which aims to prepare the health facility as an enabling environment for ANC providers to deliver FGM prevention and care services. A process evaluation will be conducted to explore ‘how’ and ‘why’ the intervention package achieves its intended results. The study design, methodology and analysis plan conform to the 2010 Consolidated Standards of Reporting Trial (CONSORT) checklist [12] while the proposed process evaluation conforms to the Consolidated Framework for Implementation Research (CFIR) recommendations [13].

**Fig. 1:**
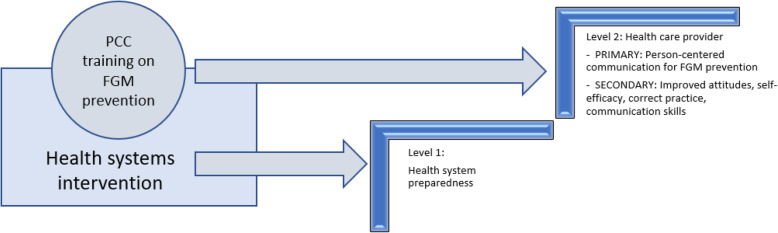
Staggered intervention implementation

The study interventions will target ANC clinics and ANC providers but will not affect the routine ANC services offered. Since we are proposing a dual testing of the effectiveness of a novel training and health systems strengthening approach, implementation of the study intervention components will follow a staggered approach (Fig. 1). The control and intervention arm will both receive Level one intervention which comprises of passive distribution of Ministry of Health directive or policy to health facilities on FGM prevention and care, WHO FGM clinical guidelines, clinical handbook on FGM and information and educational material in the form of posters to be hung to at the health facilities. The intervention arm will have ANC providers receive a novel person-centered communication training package on FGM prevention defined as the Level two intervention.

The Level one intervention will be initiated in both study arms at baseline (month 0) while the Level two intervention will be initiated at month 3 in the intervention arm only. The Level one intervention aims to improve health facility preparedness to provide FGM prevention and care services. It seeks to passively strengthen the health workforce knowledge and skills to provide treatment and care for FGM complications and to not perform FGM.

In contrast, Level two intervention is an active approach where ANC providers from the intervention sites will receive a novel training package that aims to strengthen their interpersonal communication knowledge, skills and self-efficacy to communicate in an effective, empathetic and sensitive way with their patients. The training package comprises of diverse participatory adult learning methods such as values clarification exercises; storytelling sessions; interactive games using visual training aids and handouts; and demonstrations and role plays which simulate a variety of clinical situations in which FGM is discussed as well as viewing and discussing an animated video which depicts the life of a midwife who becomes an advocate for change to end FGM.

These training activities aim to develop the person-centered skills of health care providers to enable them discuss beliefs about FGM during a clinical consultation, encourage self-reflection to challenge existent FGM beliefs and support and empower women to abandon the practice. During the training ANC providers will be trained on how to deliver a standardized “ABCD” approach for PCC i.e. “Assess” clients’ views on FGM, address and challenge their “Beliefs”, explore the possibility of “Change” and with client, “Decide” on the next steps to be taken. In doing so, it is anticipated that the patient will be empowered to own and move forward with her decisions. The delivery of this “ABCD” approach for PCC on FGM prevention will be one of the primary outcomes of the study.

### Conceptual framework

**Fig. 2:**
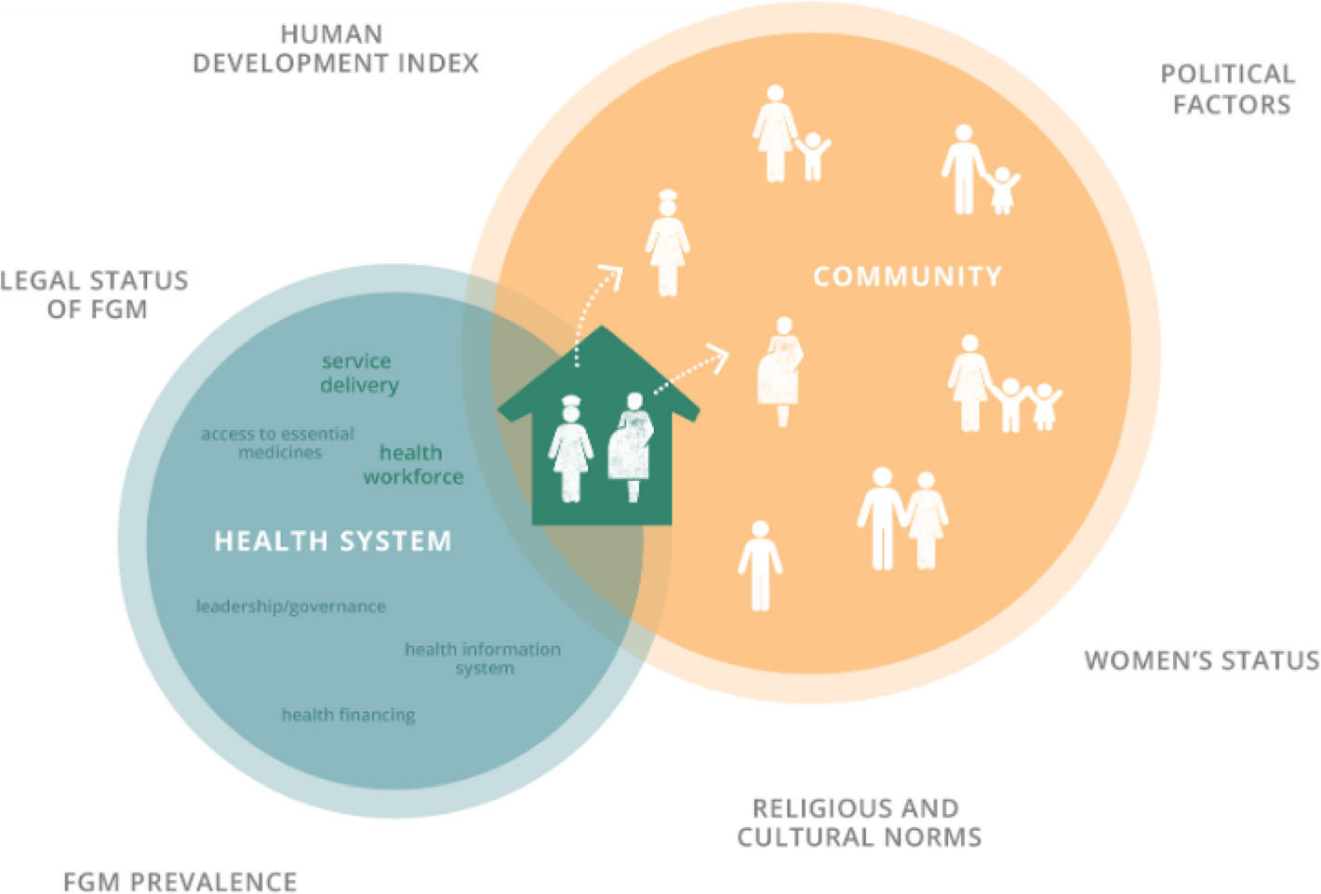
Conceptual framework/theory of change

The proposed study recognizes that health care providers are key opinion leaders in their communities who are subject to the same social norms related to FGM and that they are positioned at the intersection between the health system and the community. The study, therefore, aims to strengthen the health system while also addressing community-wide, FGM-related cultural norms by targeting the ANC providers who are mainly nurses and midwives at primary health care level (Fig. 2).

### Theoretical framework

One of the aims of the proposed intervention package is to change the values and attitudes of ANC providers towards FGM using two underlying behavioral theories. These include the Conceptualization of Attitudes Theory [14], which states that individual-level attitudes are shaped through cognition (beliefs, thoughts and understanding of a particular concept), affect (feelings and values interpretation associated with a particular concept) and past experiences (one’s previous experiences regarding a particular concept). In this study, cognition and affect will be addressed by the training on PCC for FGM prevention that will focus on changing FGM-related attitudes/values and building ANC provider skills to deliver effective FGM prevention counselling. To understand the impact of external factors in shifting individual-level attitudes and behavior change, the Social Cognitive Theory has informed development of the intervention [15]. This is a social norm theory that addresses the way individuals are influenced by their society and the ways they can in turn, influence others around them.

### Study setting and population

#### Eligibility criteria

The proposed study will be conducted in three sub-Saharan African countries with reported high FGM prevalence. Population-based studies have shown that 98% of women and girls between 15 and 49 years in Somalia, 97% in Guinea and 21% in Kenya have undergone FGM, with 20 counties in Kenya having a prevalence of more than 80% [16]. Within each study country, 2–3 sub-national administrative units (regions/counties) have been purposively selected using the eligibility criteria below:

- Regions/counties with FGM prevalence > 50% among females 15–49 years old
- Regions/counties that have > 15 ANC clinics, seeing on average 30 new ANC clients/month
- Logistical feasibility in terms of security and transport

### Sample size

The unit of study will be ANC clinics clustered by region/county and randomized to either the intervention or control arms. Each study country provided a list of government-run, primary care facilities (i.e. dispensaries and/or health centers) that offer ANC services in the selected regions/counties using Ministry of Health facility administrative records. For each, data on the average number of new ANC clients seen in November and December 2019 was used to select study sites. Facilities with an average of < 30 new ANC clients per month were excluded. The sample size was calculated based on the ability to detect a 10% difference between the intervention and control arms in the primary outcome. A similar intervention in which providers were trained to provide PCC, generated a 20% difference in another study [17]. The sample sizes in Table 1 assume that in each country, 30 ANC clinics with 300 first ANC visit clients are needed in each arm so as to have sufficient power to detect a significant difference in the study outcome of interest, if one exists, and allowing for a 10% non-response rate.

**Table 1:** Number of regions/counties, ANC sites, ANC providers and first ANC visits clients to be sampled for Randomized Control Trial

Country	Regions/Counties	# of ANC sites included	# of ANC providers included	# of first ANC clients included
Guinea	• Conakry • Faranah	Intervention: 30 Control: 30 Total: 60	Intervention: 30 Control: 30 Total: 60	Intervention: 900 Control: 600 Total: 1500
Kenya	• Kajiado • Baringo • Elgeyo Marakwet	Intervention: 30 Control: 30 Total: 60	Intervention: 30 Control: 30 Total: 60	Intervention: 900 Control: 600 Total: 1500
Somalia	• Awdal • Maroodi Jeeh • Togdheer	Intervention: 30 Control: 30 Total: 60	Intervention: 30 Control: 30 Total: 60	Intervention: 900 Control: 600 Total: 1500

### Sampling and randomization procedures

Randomization will be done at region/county level. All sampled ANC clinics will be paired based on average number of new ANC clients in November/December 2019, and then randomized into intervention and control arms on a 1:1 basis using STATA. The clinics will be blinded as to study arm allocation although ANC providers invited for the training on PCC for FGM prevention at month 3 may infer that their clinics are in the intervention arm. All ANC client participants will be blinded to study arm allocation of their ANC clinic.

For the process evaluation, sites will be selected based on responses from the month 3 data collection to identify sites that are complying with the level one intervention component. A convenience sample of six ANC providers and six new ANC clients from these clinics will be identified.

### Data collection

Data collectors in all countries will be trained by standardized training developed by WHO with WHO participation and supervision. Similarly, data collection will have regular supervision and monitoring. To ensure high participation, participating sites will be notified in advance on planned data collection dates. Data management with regards to data entry, coding, security, and storage, including any related processes to promote data quality are detailed in the study operations manual. Quantitative data will be collected at three time points in the intervention arm: baseline, month 3 and month 6; and at two time points in the control arm: baseline and month 6. All data collection will be conducted using structured questionnaires pre-programmed into hand-held tablets. Quantitative data collection instruments will include a screening tool for ANC providers to assess their socio-demographic information (SCR); a questionnaire assessing their FGM-related practices, skills, and attitudes (HCP); an exit questionnaire (EXT) administered to first ANC visit clients to assess their exposure to level one intervention components as well as their perception of and satisfaction with the ANC visit encounter as relates to counselling for FGM prevention provided through the ABCD approach (Table 2). All data collection tools will be translated into local languages, tested and validated in the three countries.

**Table 2:** Respondents and data collection tools at different study time-points

Respondent	Data Collection Tool	# to be Interviewed	Time-Point
Cluster Randomized Trial			
ANC providers	**SCR Questionnaire**	30 in each arm	**Intervention:** Baseline **Control:** Baseline
	**HCP Questionnaire**	30 in each arm	**Intervention:** 0, 3, 6 months **Control:** 0, 6 months
New ANC clients	**EXT questionnaire**	300 in each arm	**Intervention:** 0, 3, 6 months **Control:** 0, 6 months
Process Evaluation			
ANC providers	**In-depth interviews**	6	6 months
New ANC clients	**In-depth interviews**	6	6 months
Health facility	**Health facility checklist**	30 in each arm	0, 3, 6 months

A health facility assessment will be conducted at baseline, month 3 and month 6 in both intervention and control arms using a health facility checklist (CHK), which will assess compliance to the level one intervention component using an observational checklist as well as a short set of questions for health facility managers. The observation will assess dose and fidelity of the level one intervention component. The questions for the facility managers will assess catchment population, number of ANC providers, number of ANC clients seen per month, as well as number of staff meetings and supervisory visits held. It will also assess the facility’s environmental context regarding any pro- or anti-FGM activities targeting its catchment population.

Qualitative data in the form of in-depth interviews (IDIs) among a convenience sample of ANC providers and first ANC visit clients will be conducted at month 6 in the intervention clinics as described above. The IDIs will explore the acceptability, feasibility and other motivational/de-motivational factors that might influence intervention implementation. The IDIs will also be used to explore acceptability and appropriateness of the intervention as well as assessing respondents’ agency, perceptions of service delivery and any perceived impact of PCC on their FGM-related values/attitudes. The IDIs will be guided by an interview guide (Supplementary File 1).

### Data analysis

Data analysis will be conducted using both intention-to-treat and per-protocol approaches. A per-protocol analysis will utilize data from both ANC providers and their clients only if the provider was working in a clinic in the intervention arm and received the novel training on PCC for FGM prevention. Data from newly employed ANC providers in the intervention arm or from providers moving from an intervention to a control clinic will be excluded. No imputation of missing data will be performed.

A CONSORT flow chart describing excluded data and loss-to-follow up will be presented. Data analysis will be done using STATA 16 (StataCorp Inc., College Station, TX, USA) statistical analysis software.

At baseline, descriptive statistics will be used to compare client and clinic characteristics in the intervention versus control arms. Two-level regression models, with first ANC visit clients as the first level and ANC clinics as the second level, will be fitted. The ANC provider level will not be included since there is generally a low number of providers per ANC clinic in the study setting and it will not be ethical to collect data connecting provider and client. The region/county level will also be excluded given the low number of regions/counties per country and to limit the complexity of the multilevel models.

The primary study outcomes include the delivery of PCC on FGM using the “ABCD” approach by ANC providers measured from responses from validated patient and provider assessment measures that use the four constructs of the operational definition of person-centered communication [18]. As well as a health systems intervention package availability within ANC clinic and its use by ANC providers composite score defined as “health facility preparedness score” to provide FGM prevention and care services.

The secondary self-efficacy outcome will be assessed based on a score calculated from a validated tool for measuring general self-efficacy [19]. Interpersonal communication skills will be measured using a standardized questions on interpersonal communication while knowledge, attitude and practice skills on FGM prevention and care will be measured from a modified KAP questionnaire used in the formative research. Feasibility, acceptability, fidelity and coverage of interventions will be measured during the process evaluation. Unintended harms will be recorded and assessed in compliance with the study SOPs.

**Fig. 3:**
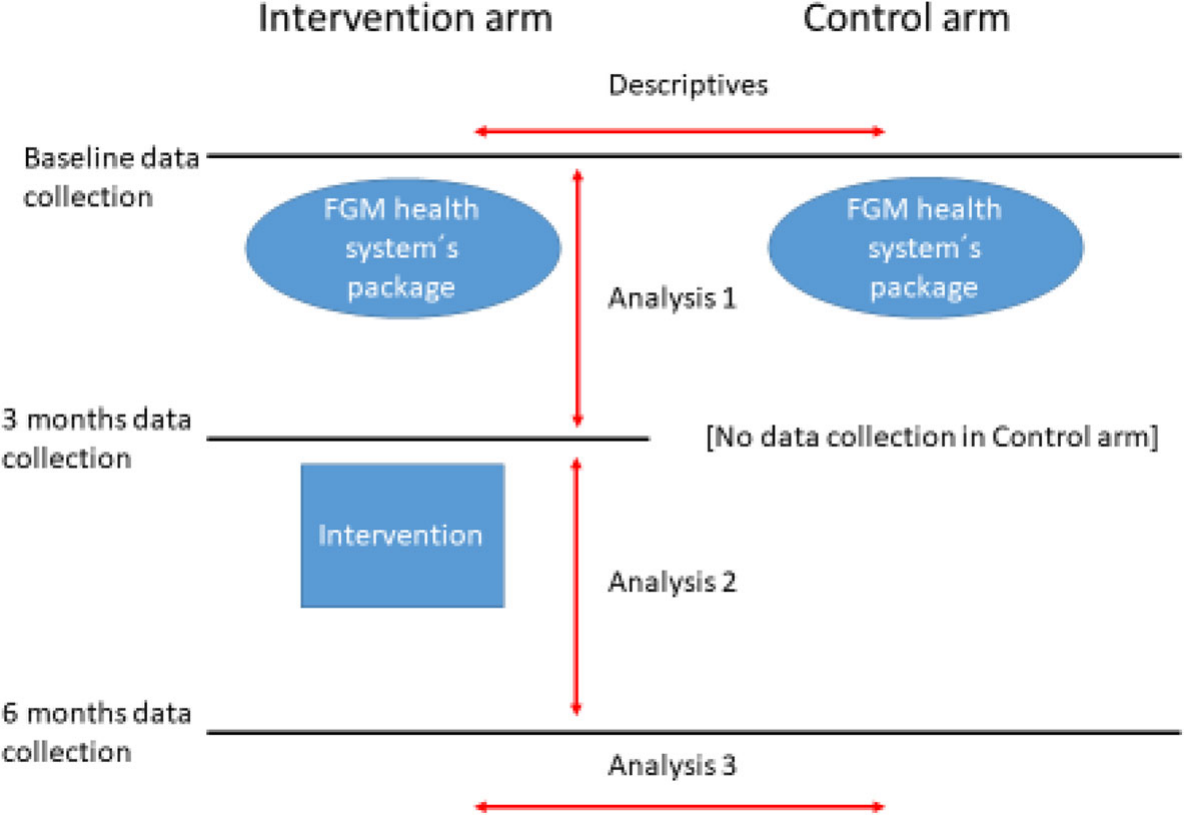
Data analysis plan

To determine the impact of the Level one intervention component, changes from baseline to month 3 in the intervention arm will be analyzed (Fig. 3). Analysis of change from month 3 to month 6 in the intervention arm will be used to determine the impact of the training on PCC for FGM prevention. At month 6, comparison of study primary, secondary and intermediary endpoints between the intervention and control arms will assess the impact of the complete intervention package. Both crude and adjusted Odds Ratios (OR) will be presented.

ANC providers (midwives/nurses) from intervention sites will be selected at month 6 of the study timeline (based on data from month 3) for in-depth interviews as part of the process evaluation. Audio recordings of the IDIs will be saved as electronic files on password-protected computers. These will be transcribed and translated into English for analysis. The transcripts will be reviewed by the in-country principal investigator (PI) to ensure data quality and content accuracy. An in-country codebook will be developed. New codes will be added, and the codebook revised accordingly upon consultation with the WHO team. Atlas.ti software (Atlas.ti version 5, Scientific Software Development GmbH, Berlin, Germany) will be used for analysis of qualitative data. As required by the WHO ethics review committee (ERC), all audio recordings will be erased within 2 years of publication of study findings, or if there is no publication, no later than 6 years after the end of the study.

### Data monitoring

Ongoing data quality and safety will be monitored by the in-country PI and data manager during data collection, entry and analysis. Periodic data audits at study initiation and thereafter, at regular intervals, will be conducted by the WHO team. There will be no data monitoring committee as research team will monitor unintended harms and planning interim analysis at 3 months. Participants who suffer unintended harms will be followed up beyond study end date.

### Ethical considerations

Ethical approval for the master protocol (25/07/2019 version 6) has been obtained from the WHO ERC (P151/03/2014). Each study country submitted country-specific protocols to local institutional review boards (IRBs). In Kenya, ethical approval was obtained from the University of Nairobi/Kenyatta National Hospital ERC (P805/09/2019), while in Somalia it was obtained from the Department of Planning, Policy and Strategic Information, Unit of Research (MOHD/DG: 2/11526/2019). The *Comite National d’Ethique Pour la Recherche en Sante* (CNERS) provided ethical approval in Guinea (105/CNERS/19). Given the illegality of FGM in two countries (Kenya and Guinea), all study participants will provide verbal informed consent. Important protocol amendments will be communicated to WHO ERC and in-country IRBs. Informed consent will be obtained from all study participants. Detailed steps to ensure participant confidentiality before, during and after study are included in the study operations manual.

### Collaborating institutions and dissemination of results

The proposed study will be coordinated by the WHO, Department of Sexual and Reproductive Health (SRH). In-country research institutions include the University of Nairobi (UoN), College of Health Sciences in Nairobi, Kenya; the Centre for Research on Reproductive Health in Guinea - *Cellule de recherche en santé de la reproduction en Guinée (CERREGUI)* in Conakry, Guinea and the Data and Research Solutions (DARS) in Hargeisa, Somalia which all have been involved in study design conceptualization and will conduct the data collection, analysis and dissemination.

The study findings will be disseminated within each country to the study participants and their respective community health committees, ministry of health, governmental and non-governmental counterparts involved in national FGM abandonment strategy, national research dissemination meetings and internationally through international meetings and publications.

## Discussion

While efforts to eradicate FGM have been ongoing for decades, there is lack of clear evidence on what works to prevent the practice and on the potential role of the health sector in this process [7]. A recent evaluation of a social norm change intervention showed promising results using community engagement strategies to address underlying social norms supportive of FGM [20]. A systematic review of health sector interventions found evidence from eight sub-Saharan Africa countries (Burkina Faso, Egypt, Ethiopia, Somalia, Kenya, Mali, Nigeria, and Senegal) on what activities and strategies the health sector could take to address FGM. These ranged from classroom didactic training, media communication, outreach and advocacy, as well as informal adult education [21]. These studies however, lacked scientific rigor; with majority using pre-and post-test assessments, lacking a comparison group or standardized measures, having follow-up periods that were too short to demonstrate impact, and not adequately controlling for contextual factors.

There is also lack of literature on the effectiveness of health system strengthening approaches towards FGM prevention. Another recent review exploring policy, training and service delivery interventions within the health sector found promising initiatives that have led to changes in attitudes among health care providers working with FGM affected populations [22]. However, there is lack of clear evidence from the health sector on what works to foster sustainable behaviour change. A systematic review of studies on the effectiveness of health systems strengthening strategies for other health issues found that educational meetings, trainings on educational outreach, practice facilitation, use of local opinion leaders, audit and feedback, and tailored interventions led to improvements in the health workforce capacity and service delivery, critical building blocks of health systems [23]. Analysis of the effect of the health system strengthening package in this study will, therefore, provide additional evidence to build on existing literature.

The training on PCC for FGM prevention developed for this study draws on existing literature on person-centred care, a key component of quality health care. Available evidence suggests that person-centred care can lead to improved clinical outcomes for patients since health providers are better able to understand patient concerns and communicate more effectively, resulting in improved patient satisfaction [24, 25].

Effective interpersonal communication skills are essential for the delivery of person-centred care. Existing evidence suggests that health care providers tend to have poor interpersonal communication skills, low self-efficacy and knowledge regarding FGM prevention and care [9, 10, 25]. Finally, the intervention package to be implemented and tested in this study builds on existing tools and knowledge, responds to country needs, draws on relevant evidence-based materials, is based in behavioural theory, while also recognizing the complexity of FGM as a social norm. For these reasons, the proposed study will generate timely scientific evidence on the effectiveness of strengthening the health systems, including building the capacity of the health workforce, towards effective FGM prevention and care.

## Supplementary Information

**Additional file 1.** Study tools and in-depth interview guides and consent model form.

**Additional file 2.** Ethical approvals for study from WHO and in-country institutional review boards for Guinea, Kenya and Somalia.

## Data Availability

Study intervention training package will be published and made available in WHO website. Countries will access their country specific data as per contractual technical agreements. Study data sets will be made available in a publicly available repository.

## Abbreviations

ANC: Antenatal Clinic
CERREGUI: *Cellule de recherche en santé de la reproduction en Guinée* (Centre for Research in Reproductive Health in Guinea)
CFIR: Consolidated Framework for Implementation Research
CONSORT: Consolidated Standards of Reporting Trials
DARS: Data and Research Solutions
ERC: Ethical Review Committee
FGM: Female Genital Mutilation
HSS: Health System Strengthening
IRB: Institutional Review Board
PCC: Person-Centered Communication
PI: Principal Investigator
SRH: Department of Sexual and Reproductive Health and Research
SDG: Sustainable Developmental Goals
UoN: University of Nairobi
WHO: World Health Organization
