# Supplementary material for: A hybrid, effectiveness-implementation research study protocol targeting antenatal care providers to provide female genital mutilation prevention and care services in Guinea, Kenya and Somalia
Source: BMC Health Serv Res. 2021 Feb 1;21:109. doi: 10.1186/s12913-021-06097-w (PMC7848669; doi:10.1186/s12913-021-06097-w)
Supplement: Supplementary file 1 — Additional file 1. Study tools and in-depth interview guides and consent model form. [file 12913_2021_6097_MOESM1_ESM.zip › Supplementary File 1/consent forms MODEL FORM.docx]

Annex 5: Informed consent forms

**5.1 Informed consent form (ICF) - ANC providers**

*This informed consent form is for* ***ANC providers*** *in study sites in* ***[country]****, who are invited to participate in the research project “***Health systems approach to prevention of FGM using Person -Centered Communication: Implementation Research Project in Guinea, Somalia and Kenya”.**

**This informed consent form (ICF) has two parts:**

• **Information sheet (to share information about the study with you)**

• **Certificate of consent (for signatures if you choose to participate)**

**You will be given a copy of the full informed consent form**

**Part I: Information Sheet**

**Introduction**

Good day. My name is _______________, and I work for __________. I am doing research on how to best support ANC providers working in health centers to provide better care to their ANC clients. I am going to give you some information and invite you to be a part of this research. If there is anything that is not clear, please ask me to explain. If you have questions later, you can ask me or another researcher.

**Purpose of the research**

The purpose of this study is to see the effect of different approaches that aim to improve the ways that health care providers discuss female genital mutilation (FGM) and provide services for health complications of FGM at primary health centers.

**Type of Research**

Your health center was selected to take part in the study. Each health center will be assigned by chance to one of two groups, which may include a training workshop and/or receipt of documents and information about FGM at the clinic. The research project will last for 6 months in total.

**Participant Selection**

You are being invited to take part in this research because you work in a health center that was selected to participate in the study.

**Voluntary Participation**

Your participation in this research is entirely voluntary. It is your choice whether to participate or not. If you choose not to participate, it will not affect your job or on any work-related reports. You can stop participating at any time.

**Procedures**

We will ask you some questions that take approximately 20 minutes on two to three occasions—today in six months from today and possibly in three months from now. The questions will include information about yourself, your knowledge about FGM, your beliefs, practice and skills. You do not have to share any information that you are not comfortable sharing. All the information collected by data collectors will be confidential and will not be linked to you. Your responses will be anonymous, and you will not be asked to provide your name.

You may also be selected by chance in month 6 to participate in an interview by the research team. This would take approximately 45 to 60 minutes. It would take place in a private location in the clinic. The interview would be tape-recorded so that the researcher could write down your responses, but no-one would be identified by name on the tape. Tapes will be kept in a locked cabinet. The information recorded is confidential, and no one else except ***[name of person(s)]*** will have access to the tapes. The tapes will be destroyed after 6 months once they have been transcribed.

**The participant understands the study purpose and study procedures.**

**Confirm with your initials in the box.**

**Risks**

There is a risk that you may be uncomfortable during the training or during the research discussing the sensitive topic of FGM. You do not have to answer any question or take part in the discussion if you feel the question(s) are too personal or if talking about them makes you uncomfortable.

**Benefits**

There will be no direct benefit to you, but your participation will help us understand more about how to improve health care for women in primary health centers like yours, which could help your community and country.

**The participant understands the risks and benefits of the study.**

**Confirm with your initials in the box.**

**Reimbursements**

You will not receive any monetary compensation for participating in this research.

**Confidentiality**

It is possible that the research may draw attention in your local community, and you may be asked questions by other people in the community. We will not be sharing information about you to anyone outside of the research team. Any information about you will have a number on it instead of your name. Only the researchers will know what your number is and we will lock that information up with a lock and key. It will not be shared with or given to anyone.

**The participant understands that their participation is confidential.**

**Confirm with your initials in the box.**

**Sharing the Results**

We will ask you not to talk to people outside about this research until the study is completed. Each health center and community health committee (if applicable) will receive a summary of the study results. We will also publish the results so that other interested people may learn from the research.

**Who to Contact**

If you have any questions, you can ask them now or later. If you wish to ask questions later, you may contact any of the following:

***[name, address/telephone number/e-mail]***

*Example of question to elucidate understanding:* ***Do you know that you do not have to take part in this study if you do not wish to? You can say No if you wish to? Do you know that you can ask me questions later, if you wish to? Do you know that I have given the contact details of the person who can give you more information about the study? Etc.***

You can ask me additional questions about any part of the research study, if you wish to. Do you have any questions?

Part II: Verbal Consent

**The participant has been read the foregoing information and had the opportunity to ask questions.**

**Confirm with your initials in the box.**

**Confirm all checkboxes are completed.**

**Confirm with your initials in the box.**

**The participant consents to participate in the study.**

**Confirm with your initials in the box.**

**(If relevant) Confirm if participant agrees to participate in the in-depth interview.**

**Confirm with your initials in the box.**

**Statement by the researcher/person taking consent.**

**I have accurately read out the information sheet to the potential participant, and to the best of my ability made sure that the participant understands that the following will be done:**

**I confirm that the participant was given an opportunity to ask questions about the study, and all the questions asked by the participant have been answered correctly and to the best of my ability. I confirm that the individual has not been coerced into giving consent, and the consent has been given freely and voluntarily.**

**A copy of this ICF has been provided to the participant unless the participant refused to take the ICF.**

**Print name of researcher****/person taking the consent________________________**

**Signature of researcher /person taking the consent__________________________**

**Date ___________________________**

Day/month/year
