# Supplementary material for: A hybrid, effectiveness-implementation research study protocol targeting antenatal care providers to provide female genital mutilation prevention and care services in Guinea, Kenya and Somalia
Source: BMC Health Serv Res. 2021 Feb 1;21:109. doi: 10.1186/s12913-021-06097-w (PMC7848669; doi:10.1186/s12913-021-06097-w)
Supplement: Supplementary file 1 — Additional file 1. Study tools and in-depth interview guides and consent model form. [file 12913_2021_6097_MOESM1_ESM.zip › Supplementary File 1/A65933DatForms_Tools_V1.0_24Feb2020.pdf]

| World Health Organization | A Health Systems Approach to Prevention of Female Genital Mutilation using Person-Centered Communication: A Multi-Country Implementation Research Project<br>**Health Facility Checklist (CHK)** | CHK<br>Page 1/1<br>V1.0 (24Feb2020) |
|---|---|---|

**Project ID** A 6 5 9 9 3 **Centre ID** ____ - **Region/County ID** __ - **Facility ID** ___

**Study period:** ☐
0 = Baseline, 2 = Six Months

## SECTION A - FACILITY OBSERVATION

***Check around the facility for the following:***

1. Is there an MoH policy on FGM posted on the wall? ☐
   1 = Yes
   2 = No
   1a) If ***Yes***, is it placed where health care providers can see/read it e.g. bulletin board? ☐
   1 = Yes 2 = No

2. Are there WHO FGM prevention posters on the wall of the waiting room? ☐
   1 = Yes 2 = No
   2a) If ***Yes***, are they placed in a place where ANC clients can see them? ☐
   1 = Yes 2 = No

3. Is there a WHO FGM Clinical Handbook in the ANC consultation room? ☐
   1 = Yes 2 = No
   3a) If ***Yes***, is it placed where ANC providers can see it/use it? ☐
   1 = Yes 2 = No

4. Is there an FGM ABCD guide in the ANC consultation room? ☐
   1 = Yes 2 = No
   4a) If ***Yes***, is it placed where ANC providers can see it/use it? ☐
   1 = Yes 2 = No

## SECTION B - FACILITY FUNCTIONALITY

***The following questions should be directed to the health facility manager.***
***I am going to ask you a few questions about this health facility***

5. Number of ANC providers: ☐

6. Average number of ANC clients per month: ☐☐☐

7. Number of ANC providers who received training as part of the study ☐
   1 = All
   2 = Some
   3 = None (***Skip to Q8***)
   7a) If ***All*** or ***Some***, specify number trained ☐

8. Number of MoH supervisory visits to the clinic in the past year: ☐☐

9. How frequently are staff meetings held? ☐
   1 = Monthly
   2 = Every 2 to 4 months
   3 = Every 6 to 12 months
   4 - More than 12 months
   5 = Never

10. What is the size of the population served by this facility? (***specify number***) ☐☐☐☐

## SECTION C - FACILITY CONTEXT

***I am going to ask you about anti or pro FGM activities in the area served by this health facility***

11. Are there anti-FGM activities that target the population served by this health facility? ☐
    1 = Yes
    2 = No
    3 = I don't know

    11a) If ***Yes***, specify: ____________________

    ____________________

12. Are there pro-FGM activities that target the population served by this health facility? ☐
    1 = Yes
    2 = No
    3 = I don't know

    12a) If ***Yes***, specify: ____________________

    ____________________

    ____________________

## COMMENTS

____________________

***Data Collector name:*** ***Signature:***

____________________

***Date:***

| Day | | Month | | Year | | | |
|---|---|---|---|---|---|---|---|
| | | | | | | | |

***Time questionnaire completed (00:00 - 23:59):*** ☐☐ : ☐☐
hours minutes

| World Health Organization | A Health Systems Approach to Prevention of Female Genital Mutilation using Person-Centered Communication: A Multi-Country Implementation Research Project<br>**ANC Provider Screening Questionnaire (SCR)** | SCR<br>Page 1/1<br>V1.0 (24Feb2020) |
|---|---|---|

**Project ID** A 6 5 9 9 3 **Centre ID** ☐☐☐☐ - **Region/ County ID** ☐ - **Facility ID** ☐☐☐ **Provider ID** - ☐

## SECTION A - SOCIO-DEMOGRAPHIC INFORMATION

***I am going to ask you a few questions about yourself***

1. How old are you (years)? ☐☐

2. What is your sex? (***Observe and document*** ) ☐
   1 = Female
   2 = Male

***For Somali study site, skip to Q4***

3. What is your religion? ☐
   0 = None
   1 = Muslim
   2 = Christian
   3 = Other
   4 = Refused to answer
   3a) If ***Other*** , specify: ____________

4. What is the highest education level you achieved? ☐
   1 = Certificate
   2 = Diploma
   3 = Bachelors
   4 = Masters or above
   5 = Other
   4a) If ***Other*** , specify: ____________

5. What is your current professional title? ☐
   1 = Midwife 3 = Nurse - Midwife
   2 = Nurse 4 = Other
   5a) If ***Other*** , specify: ____________

6. For how many years have you been working in your current professional title? ☐☐

## SECTION B - TRAINING

***Now, I am going to ask you a few questions about specific trainings you may have received***

7. During your clinical training, did you receive any formal training on female genital mutilation? ☐
   1 = Yes 3 = I don't know (***Skip to Q10*** )
   2 = No (***Skip to Q10*** )

8. When did you receive this training? ☐
   1 = During my studies (pre-service training)
   2 = After graduation/at work (in-service training)
   3 = Both options 1 and 2

9. What was the format of this training?
   1 = Yes 3 = I don't know
   2 = No
   9a) Classroom lessons ☐
   9b) Workshops ☐

**9.** ***Continued***
   1 = Yes 3 = I don't know
   2 = No
   9c) Digital format (E-learning videos; smart phone app) ☐
   9d) During clinical practice under supervision of a mentor ☐
   9e) Other ☐
   9es) If ***Other*** , specify: ____________

10. During your pre- or post- graduate training, did you receive any formal training on communication or counselling? ☐
   1 = Yes 3 = I don't know
   2 = No

11. During you pre or post graduate training, did you receive any formal training on person-centered care? ☐
   1 = Yes 3 = I don't know
   2 = No

## SECTION C - FGM HISTORY

***Now, I will ask you a few personal questions about FGM***

***If Male provider, Skip to Q13***

12. Many women in your community have had their genitals cut when they were children. If you are comfortable telling me, can I ask if you have undergone this practice? ☐
   1 = Yes 3 = I don't know
   2 = No 4 = Refused to answer

13. Have you ever cut the genitals of a girl or a woman for non-health reasons? ☐
   1 = Yes 3 = Refused to answer
   2 = No
   13a) If ***Yes*** , have you ever cut a girl <18 years? ☐
   1 = Yes 3 = Refused to answer
   2 = No

## COMMENTS

____________

***Data Collector name:*** ***Signature:***

____________

***Date:***

| Day | | Month | | Year | | | |
|---|---|---|---|---|---|---|---|
| | | | | | | | |

***Time questionnaire completed (00:00 - 23:59):*** ☐☐ : ☐☐
**hours** **minutes**

| Project ID | Centre ID | Region/ County ID | Facility ID | Provider ID | Study period: |
|---|---|---|---|---|---|
| A 6 5 9 9 3 | | - | - | - | 0 = Baseline, 1 = Three months, 2 = Six Months |

## SECTION A - FGM KNOWLEDGE

*I am going to ask you a few questions regarding FGM*

1. Have you ever heard about female genital mutilation?
   1 = Yes
   2 = No

2. Do the women in your community undergo female genital mutilation?
   1 = Yes	3 = I don't know
   2 = No

3. Do you know of the WHO classification for female genital mutilation?
   1 = Yes
   2 = No (***Skip to Q5*** )

4. Please provide the WHO classification for the following FGM images (***to include images*** )
   1 = Type I	4 = Type IV
   2 = Type II	5 = I don't know
   3 = Type III	6 = Other

   4a) IMAGE of Type IV FGM to be inserted here
   4as) If ***Other*** , specify: ____________

   4b) IMAGE of Type I FGM to be inserted here
   4bs) If ***Other*** , specify: ____________

   4c) IMAGE of Type II FGM to be inserted here
   4cs) If ***Other*** , specify: ____________

   4d) IMAGE of Type III FGM to be inserted here
   4ds) If ***Other*** , specify: ____________

5. Do you know of any health complications arising from female genital mutilation?
   1 = Yes	2 = No

6. Is female genital mutilation illegal in your country ***(specify actual study country)?***
   1 = Yes	3 = I don't know
   2 = No

7. Are you aware of any existing WHO tools/guidance on FGM prevention and care?
   1 = Yes	2 = No
   7a) If ***Yes*** , please specify: ____________

   ____________

8. When you treat or attend to a girl or a woman with female genital mutilation, how confident are you that you have enough knowledge to provide good quality health care?
   1 = Not confident
   2 = Somewhat confident
   3 = Confident

9. How confident are you in your knowledge to communicate on FGM prevention?
   1 = Not confident
   2 = Somewhat confident
   3 = Confident

10. Would you like to receive more training related to care for women and girls with FGM?
   1 = Yes	2 = No

11. Would you like to receive more training on how to help patients prevent FGM?
   1 = Yes	2 = No

## SECTION B - FGM ATTITUDE

***For each of the following statements please state if you agree/disagree or don't know.***
1 = Agree	3 = I Don't know
2 = Disagree

12. A girl who has not undergone FGM is unclean

13. A girl who has not undergone FGM cannot be married within her community

14. A girl who has not undergone FGM is a disgrace to her family's honour

15. Health care providers who perform FGM are violating medical ethics

16. Health care providers who perform FGM should be punished

17. FGM is a good practice

18. FGM is a violation of women's and girls' rights

19. FGM is a religious mandate

| Project ID | Centre ID | Region/ County ID | Facility ID | Provider ID | Study period: |
|---|---|---|---|---|---|
| A 6 5 9 9 3 | | - | - | - | 0 = Baseline, 1 = Three months, 2 = Six Months |

## SECTION C - FGM PRACTICE

***Now, I am going to ask what you will do in specific situations regarding FGM***

20. Pretend you had a daughter now who was at an age when cutting occurs, what would your intention to cut her be?
    1 = Intend to cut her
    2 = Do not intend to cut her
    3 = I don't know
    4 = Refused to answer

21. If a family brought their daughter to the clinic requesting genital cutting for non-health reasons, would you perform it?
    1 = Yes
    2 = No
    3 = I don't know
    4 = Refused to answer

22. How often do you discourage a pregnant woman expecting to have a girl, or one having a girl at the age of cutting, from having her daughter cut?
    1 = Always
    2 = Often
    3 = Sometimes
    4 = Rarely
    5 = Never

23. If you became aware of a colleague performing female genital mutilation, will you …
    1 = Yes
    2 = No
    3 = I don't know

    23a) Report him/her?

    23b) Explain to him/her that health care providers should not perform female genital mutilation?

24. How often do you look for female genital mutilation when performing a gynecological examination of the vulva?
    1 = Always    4 = Rarely
    2 = Often    5 = Never
    3 = Sometimes

25. How often do you record female genital mutilation in the woman's medical file if you are aware that she has undergone FGM?
    1 = Always    4 = Rarely
    2 = Often    5 = Never
    3 = Sometimes

## SECTION D - CONFIDENCE

***Now I would like to ask you a few questions about how you solve problems that you face. Please tell me how much you agree or disagree with the statements that I read to you***

1 = Strongly disagree
2 = Disagree
3 = Neither agree nor disagree
4 = Agree
5 = Strongly agree

26. I will be able to achieve most of the goals that I have set for myself

27. When facing difficult tasks, I am certain that I will accomplish them

28. In general, I think that I can obtain outcomes that are important to me

29. I believe I can succeed at almost any endeavour to which I set my mind

30. I will be able to successfully overcome many challenges

31. I am confident that I can perform effectively on many different tasks

32. Compared to other people, I can do most tasks very well

33. Even when things are tough, I can perform quite well

| Project ID | Centre ID | Region/ County ID | Facility ID | Provider ID | Study period: |
|---|---|---|---|---|---|
| A 6 5 9 9 3 | | - | - | - | 0 = Baseline, 1 = Three months, 2 = Six Months |

## SECTION E - COMMUNICATION SKILLS

***Now, I will ask you questions about your communication skills***

34. I can put myself in others' shoes ☐
    1 = Always
    2 = Often
    3 = Sometimes
    4 = Rarely
    5 = Never

35. I let others know I understand what they say ☐
    1 = Always
    2 = Often
    3 = Sometimes
    4 = Rarely
    5 = Never

36. In conversations with my colleagues, I perceive not only what they say but what they don't say ☐
    1 = Always
    2 = Often
    3 = Sometimes
    4 = Rarely
    5 = Never

37. I communicate effectively ☐
    1 = Always
    2 = Often
    3 = Sometimes
    4 = Rarely
    5 = Never

38. I communicate with others as though they are my equals ☐
    1 = Always
    2 = Often
    3 = Sometimes
    4 = Rarely
    5 = Never

## SECTION F - HEALTH FACILITY READINESS

***These next questions relate to your clinic setting:***

39. Have you seen any FGM posters at the clinic? ☐
    1 = Yes
    2 = No

40. Have you referred to the WHO Clinical Handbook on FGM? ☐
    1 = Yes
    2 = No, available but not referred
    3 = No, not available

41. Do you think it is feasible to provide FGM prevention counselling during ANC visits? ☐
    1 = Yes
    2 = No

## COMMENTS

______________________________

______________________________

______________________________

______________________________

______________________________

______________________________

***Data Collector name:*** ***Signature:***

______________________________

***Date:***

| Day | Month | Year |
|---|---|---|
| | | |

***Time questionnaire completed (00:00 - 23:59):*** ☐☐ hours : ☐☐ minutes

# First ANC Client Exit Questionnaire (EXT)

Project ID: A 6 5 9 9 3 | Centre ID: ____ - Region/ County ID: __ - Facility ID: ___ - Participant ID: ____

Study period: ☐
0 = Baseline, 1 = Three months, 2 = Six Months

## SECTION A - SOCIO-DEMOGRAPHIC INFORMATION

*I am going to ask you a few personal questions*

1. How old are you (years)? ☐☐

***For Somali study sites, skip to Q3***

2. What is your religion? ☐
   - 0 = None
   - 1 = Muslim
   - 2 = Christian
   - 3 = Other
   - 4 = Refused to answer
   - 2a) If **Other**, specify: ______________

3. What is the highest level of education you achieved? ☐
   - 0 = None
   - 1 = Primary
   - 2 = Secondary
   - 3 = University
   - 4 = Other
   - 3a) If **Other**, specify: ______________

## SECTION B - CLINIC EXPERIENCE

***The following questions relate to your clinic visit today.***
***During your visit today:***

1 = Yes
2 = No
3 = I don't know

4. Did you see any FGM poster(s) in the waiting room? ☐
5. Did the ANC provider ask if you have undergone FGM? ☐
6. Did the ANC provider explain how FGM can harm your health? ☐
7. Did the ANC provider ask about your personal belief regarding FGM? ☐
8. Did the ANC provider discuss why FGM should be prevented? ☐
9. Did the ANC provider discuss how FGM could be prevented? ☐
10. Did you have any questions about FGM to ask the ANC provider? ☐
11. Did you feel encouraged to ask questions about FGM? ☐
12. Are you satisfied with how FGM was addressed during your visit with your ANC provider today? ☐

## SECTION C - FGM ATTITUDE AND PRACTICE

***Now, I am going to ask your opinions regarding FGM***

13. What do you feel about FGM now as compared to before you came to the clinic today? ☐
    - 1 = Same, no change
    - 2 = I feel more supportive of FGM now as compared to before I came
    - 3 = I feel less supportive of FGM now as compared to before I came
    - 4 = I don't know
    - 5 = Other
    - 13a) If **Other**, specify: ______________

14. How supportive are you of female genital mutilation? ☐
    - 1 = Strongly opposed
    - 2 = Somewhat opposed
    - 3 = Neutral (neither opposed nor supportive)
    - 4 = Somewhat supportive
    - 5 = Strongly supportive

15. Many women in your community have had their genitals cut when they were children. If you are comfortable telling me, can I ask if you have undergone this practice? ☐
    - 1 = Yes
    - 2 = No
    - 3 = I don't know
    - 4 = Refused to answer

16. Pretend you had a daughter now who was at an age when cutting occurs, what would your intention to cut her be? ☐
    - 1 = Intend to cut her
    - 2 = Do not intend to cut her (***Skip to Q18*** )
    - 3 = I don't know (***Skip to Q18*** )
    - 4 = Refused to answer (***Skip to Q18*** )

17. If Intending to cut, who would you prefer to do the cutting?
    - 1 = Traditional practitioner
    - 2 = Health care provider
    - 3 = Other
    - 17a) If **Other**, specify: ______________

18. Do you wish/want to be active in preventing FGM? ☐
    - 1 = Yes
    - 2 = No
    - 3 = I don't know

## COMMENTS

***Data Collector name:*** ______________ ***Signature:*** ______________

***Date:***

| Day | | Month | | Year | | | |
|---|---|---|---|---|---|---|---|
| | | | | | | | |

***Time questionnaire completed (00:00 - 23:59):*** ☐☐ hours : ☐☐ minutes
