# Supplementary material for: A hybrid, effectiveness-implementation research study protocol targeting antenatal care providers to provide female genital mutilation prevention and care services in Guinea, Kenya and Somalia
Source: BMC Health Serv Res. 2021 Feb 1;21:109. doi: 10.1186/s12913-021-06097-w (PMC7848669; doi:10.1186/s12913-021-06097-w)
Supplement: Supplementary file 1 — Additional file 1. Study tools and in-depth interview guides and consent model form. [file 12913_2021_6097_MOESM1_ESM.zip › Supplementary File 1/A65933DatForms_Interviews_V1.0_24Feb2020.pdf]

IN-DEPTH INTERVIEW TOPIC GUIDE (ANC CLIENTS)

Project ID	Centre ID	Region/ County ID	Facility ID
A 6 5 9 9 3		-	-

Step 1: Introduce yourself to the interviewee. Describe the purpose of the interview and how the information will be used. Obtain verbal consent.

Step 2: Ask the interviewee to complete the information below on their socio-demographic information prior to beginning the interview.

Step 3: Conduct the interview. Please remember to audio-record the interview. Where applicable, enter your comments/observations in the lines provided after each question.

Socio-Demographic Characteristics of Interviewee

Age: ______

Marital status: ______

Highest level of education: ______

Number of children: ______

Religion *(For Somaliland interviewees, skip this):* ______

In-depth Interview Guiding Questions

Interview date: ______

Start time: ______

End time: ______

1. Can you tell me what you know about FGM? ______

2. How common is FGM in your community? How has this changed from when you were a child? ______

3. Did you discuss FGM with your ANC provider today? If yes, what did you discuss? ______

4. Do you think that the nurse/midwife listened to you about your opinion on FGM? Please explain ______

Project ID	Centre ID	Region/ County ID	Facility ID
A 6 5 9 9 3		-	-

5. Do you think that the discussion with the nurse/midwife made you feel more or less supportive of FGM? Please explain.

___

___

6. How could these discussions be improved?

___

___

7. Who makes decisions about FGM in your household? What is your role in decision-making about FGM for your children?

___

___

8. How satisfied were you with the care you received today? (Probe about counselling in particular)

___

___

9. Do you think the discussion on FGM was appropriate?

___

___

10. Do you think the nurse/midwife is the right person to do this?

___

___

11. Do you think this discussion should be continued in ANC or elsewhere? Please explain.

___

___

Data Collector/interviewer's name: ______________ ***Signature:*** ______________

Date of the interview:

Day		Month		Year			

IN-DEPTH INTERVIEW TOPIC GUIDE (ANC PROVIDERS)

Project ID	Centre ID	Region/ County ID	Facility ID
A 6 5 9 9 3	☐☐☐☐ -	☐ -	☐☐☐

Step 1: Introduce yourself to the interviewee. Describe the purpose of the interview and how the information will be used. Obtain verbal consent

Step 2: Ask the interviewee to complete the information below on their socio-demographic information prior to beginning the interview

Step 3: Conduct the interview. Please remember to audio-record the interview. Where applicable, enter your comments/observations in the lines provided after each question.

Socio-Demographic Characteristics of Interviewee

Age: ____________________

Sex: ____________________

Highest level of education: ____________________

Professional title: ____________________

Number of years of experience in the health facility: ____________________

Number of years of experience in total: ____________________

Religion *(For Somaliland interviewees, skip this)* : ____________________

In-depth Interview Guiding Questions

Interview date: ____________________

Start time: ____________________

End time: ____________________

1. In general, how do your patients view the practice of FGM? ____________________

____

____

2. How do you feel about discussing FGM with your patients? ____________________

____

____

3. Do you recall the ABCD's of FGM prevention counselling? Can you describe those steps to me? ____________________

____

____

____

Project ID	Centre ID	Region/ County ID	Facility ID
A 6 5 9 9 3	-	-	

4. Are you able to carry out the ABCD steps? What is working and what is not working? How can it work better?

5. Can you describe a discussion on FGM with a patient that went well?

6. Can you describe a discussion on FGM with a patient that was difficult? How did you handle the situation?

7. How easy or difficult would it be to use ABCD steps to communicate about FGM prevention with patients attending other services, including immunization services?

8. What do you feel can help you to provide FGM prevention?

9. What do you feel makes it difficult to provide FGM prevention?

Project ID	Centre ID	Region/ County ID	Facility ID
A 6 5 9 9 3	☐☐☐☐ -	☐ -	☐☐☐

10. Do you think having posters or policies or job aids facilitate the implementation of ABCD's of FGM prevention? Explain How?

____

____

____

11. Do you think the training you received was useful? How? Why?

____

____

____

12. How can discussions with patients on FGM be done better (facilitating factors)

____

____

____

13. Do you believe discussions with antenatal patients will make any difference to the practice of FGM in the community?

____

____

____

Data Collector/interviewer's name: ______________________ ***Signature:*** ______________

Date of the interview:

Day		Month		Year			
