# Supplementary material for: A hybrid, effectiveness-implementation research study protocol targeting antenatal care providers to provide female genital mutilation prevention and care services in Guinea, Kenya and Somalia
Source: BMC Health Serv Res. 2021 Feb 1;21:109. doi: 10.1186/s12913-021-06097-w (PMC7848669; doi:10.1186/s12913-021-06097-w)
Supplement: Supplementary file 2 — Additional file 2. Ethical approvals for study from WHO and in-country institutional review boards for Guinea, Kenya and Somalia. [file 12913_2021_6097_MOESM2_ESM.zip › Supplementary File 2/Guinea- Accord Comite Ethique.pdf]

REPUBLIQUE DE GUINEE

===========

TRAVAIL – JUSTICE – SOLIDARITE

COMITE NATIONAL D'ETHIQUE POUR LA RECHERCHE EN SANTE (CNERS)

Conakry, le 20 Novembre 2019

N° : 105/CNERS/19
Objet : Examen protocole

LA PRESIDENTE
Au Prof. Baldé Mamadou Djouldé
Conakry, Hamdallaye
Email: baldemddka@gmail.com

Monsieur,

Le Comité National d'Ethique pour la Recherche en Santé (CNERS) a procédé à l'examen de la version corrigée de votre protocole intitulé : **Une approche des systèmes de santé pour la prévention des mutilations génitales féminines utilisant un système de communication centrée sur les personnes : Projet de recherche sur la mise en œuvre en Guinée, en Somalie et au Kenya. – Application en Guinée**

Le CNERS prend note des corrections et des clarifications apportées à la première version de ce protocole.

Le CNERS autorise la mise en œuvre de votre protocole dans le respect des principes éthiques y énoncés. Il tient à être informé de toute autre modification du présent protocole au cours de sa mise en œuvre. Il vous invite à lui transmettre le rapport final de l'étude.

Cette approbation est valable pour une période d'un an à compter de sa date de signature.

Veuillez agréer l'expression de ma considération distinguée.

COMITE NATIONAL D'ETHIQUE POUR LA RECHERCHE EN SANTE * C.N.E.R.S - GUINEE * La PRESIDENTE

La Présidente P.O

Dr Ousmane SOUARE

Présidente : Professeur Oumou Younoussa SOW. Tel. +224 664 962 434 Email : oumou45@yahoo.fr
Assistante Administrative : Aissatou BAH. Tel. +224 622 253 127 Email : aissatou_dalaba07@yahoo.fr
