# Supplementary material for: A hybrid, effectiveness-implementation research study protocol targeting antenatal care providers to provide female genital mutilation prevention and care services in Guinea, Kenya and Somalia
Source: BMC Health Serv Res. 2021 Feb 1;21:109. doi: 10.1186/s12913-021-06097-w (PMC7848669; doi:10.1186/s12913-021-06097-w)
Supplement: Supplementary file 2 — Additional file 2. Ethical approvals for study from WHO and in-country institutional review boards for Guinea, Kenya and Somalia. [file 12913_2021_6097_MOESM2_ESM.zip › Supplementary File 2/Somalila-ERC approval.pdf]

Jamhuuriyadda Somaliland

Republic of Somaliland

Wasaaradda Horumarinta Caafimaadka

Ministry of Health Development

Agaasimaha Guud

The Director General

Ref:MOHD/DG: 2/11526 /2019

Date: 24/9/2019

To: DATA AND RESEARCH SOLUTIONS (DARS)

Cc: Director of Planning Policy and strategic information MOHD

Cc: Vice Minister of MOHD

Cc: Minister of MOHD

SUBJECT: Health systems approach to prevention of Female Genital mutilation (FGM) using Person –Cantered Communication: Implementation Research Project in Somaliland

The ministry of health Somaliland, under the process of the department of planning/policy and strategic information, unit of research has reviewed and discussed your proposal documents of "Health systems approach to prevention of Female Genital Mutation using Person –Cantered Communication: Implementation Research Project in Somaliland' has been approved to conduct Maroodi-Jeeh, Awdal and Togdher regions.

REPUBLIC OF SOMALILAND * MINISTRY OF HEALTH DEVELOPMENT * JAMHUURIYADDA SL * WASAARADDA HORUMARINTA CAAFIMAADKA

Tell: +252 63 4466061 Email: herrgeye@gmail.com

1	Principal of Investigator	Data and Research Solutions (**DARS**)
2	Name of the document	Health systems approach to prevention of Female Genital Mutation using Person –Cantered Communication: Implementation Research Project in Somaliland
3	Questionnaires	Accepted/approved
4	Study proposal/protocol	Accepted and approved

We approved the study to be conducted in the presented form, the Ministry of Health Development Somaliland also expects to informed if progress or changes occurring during the study, final report (soft as well as hard copy will also be submitted to the ministry of Health Development)

NB: Custodial of the data is owned by the ministry Development

Best regard

Dr. Mohamed Abdi Hergeye

Director General of MOHD
