# Supplementary material for: A hybrid, effectiveness-implementation research study protocol targeting antenatal care providers to provide female genital mutilation prevention and care services in Guinea, Kenya and Somalia
Source: BMC Health Serv Res. 2021 Feb 1;21:109. doi: 10.1186/s12913-021-06097-w (PMC7848669; doi:10.1186/s12913-021-06097-w)
Supplement: Supplementary file 2 — Additional file 2. Ethical approvals for study from WHO and in-country institutional review boards for Guinea, Kenya and Somalia. [file Kenya - APPROVAL LETTER ERC.pdf › Supplementary File 2/Kenya - APPROVAL LETTER ERC.pdf]

UNIVERSITY OF NAIROBI
COLLEGE OF HEALTH SCIENCES
P O BOX 19676 Code 00202
Telegrams: varsity
Tel:(254-020) 2726300 Ext 44355

KNH-UON ERC
Email: uonknh_erc@uonbi.ac.ke
Website: http://www.erc.uonbi.ac.ke
Facebook: https://www.facebook.com/uonknh.erc
Twitter: @UONKNH_ERC https://twitter.com/UONKNH_ERC

KENYATTA NATIONAL HOSPITAL
P O BOX 20723 Code 00202
Tel: 726300-9
Fax: 725272
Telegrams: MEDSUP, Nairobi

Ref: KNH-ERC/A/405

29th October, 2019

KENYATTA NATIONAL HOSPITAL
APPROVED
29 OCT 2019
KNH/UoN-ERC
P. O. BOX 20723 - 00202 NAIROBI

Prof. Patrick Ndavi
County Principal Investigator
Dept. of Obstetrics and Gynecology
School of Medicine
College of Health Sciences
University of Nairobi

Dear Prof. Ndavi,

RESEARCH PROPOSAL: A HEALTH SYSTEMS APPROACH TO PREVENTION OF FGM USING PERSON-CENTERED COMMUNICATION; IMPLEMENTATION RESEARCH PROJECT IN GUINEA, SOMALIA AND KENYA (A-ID: A65993) (P805/09/2019)

This is to inform you that the KNH- UoN Ethics & Research Committee (KNH- UoN ERC) has reviewed and **approved** your above research proposal. The approval period is 29th October 2019 – 28th October 2020.

This approval is subject to compliance with the following requirements:

a. Only approved documents (informed consents, study instruments, advertising materials etc) will be used.
b. All changes (amendments, deviations, violations etc.) are submitted for review and approval by KNH-UoN ERC before implementation.
c. Death and life threatening problems and serious adverse events (SAEs) or unexpected adverse events whether related or unrelated to the study must be reported to the KNH-UoN ERC within 72 hours of notification.
d. Any changes, anticipated or otherwise that may increase the risks or affect safety or welfare of study participants and others or affect the integrity of the research must be reported to KNH- UoN ERC within 72 hours.
e. Clearance for export of biological specimens must be obtained from KNH- UoN ERC for each batch of shipment.
f. Submission of a request for renewal of approval at least 60 days prior to expiry of the approval period. (*Attach a comprehensive progress report to support the renewal*).
g. Submission of an *executive summary* report within 90 days upon completion of the study.
This information will form part of the data base that will be consulted in future when processing related research studies so as to minimize chances of study duplication and/ or plagiarism.

For more details consult the KNH- UoN ERC website **http://www.erc.uonbi.ac.ke**

Yours sincerely,

PROF. M. L. CHINDIA
SECRETARY, KNH-UoN ERC

c.c. The Principal, College of Health Sciences, UoN
The Director, CS, KNH
The Chairperson, KNH- UoN ERC
The Assistant Director, Health Information, KNH
Co-investigator: Prof. Zahida Qureshi (UoN)
Collaborators: Dr. Mamadou Dioulde Baldé (Guinea), Ahmed Diriye (Somalia), Dr. Anne Marie Soumah, Muna Abdi Ahmed
