# Supplementary material for: A hybrid, effectiveness-implementation research study protocol targeting antenatal care providers to provide female genital mutilation prevention and care services in Guinea, Kenya and Somalia
Source: BMC Health Serv Res. 2021 Feb 1;21:109. doi: 10.1186/s12913-021-06097-w (PMC7848669; doi:10.1186/s12913-021-06097-w)
Supplement: Supplementary file 2 — Additional file 2. Ethical approvals for study from WHO and in-country institutional review boards for Guinea, Kenya and Somalia. [file WHO-HQ-ERCapproval.pdf › Supplementary File 2/WHO-HQ-ERCapproval.pdf]

20, AVENUE APPIA – CH-1211 GENEVA 27 – SWITZERLAND – HTTP://WWW.WHO.INT/ETHICS/REVIEW-COMMITTEE/EN/ – HTTPS://EXTRANET.WHO.INT/ERCWEB/LOGIN.PHP

WHO ERC
Review Summary

Protocol ID: A65993
Country: Master protocol
Protocol Title: A Health systems approach to prevention of FGM using Person-Centered Communication: Implementation Research Project in Guinea, Somalia and Kenya
Version: 4 **Dated:** 23/05/2019
WHO Responsible Staff Member: Christina Catherine Pallitto
Responsible Unit: WHO/HQ/FWC/RHR
Meeting Date: 13/06/2019

Dear Dr. Christina Catherine Pallitto,
Please find the review summary of the Protocol "A Health systems approach to prevention of FGM using Person-Centered Communication: Implementation Research Project in Guinea, Somalia and Kenya", which was submitted to the Secretariat on 23/05/2019. This proposal underwent regular review.

The outcome of the review is provided below. When responding, please submit the following:

1. A cover memorandum that addresses your responses, POINT BY POINT, to each of the queries in sections A and B.
   Section C contains Suggestions to improve the proposal but there is no obligation to follow them.

2. An Amended protocol including the responses in bold, highlighted or in track changes. Please ensure that tracking formatting changes is switched off or that all formatting changes have been accepted and that no comments which the team may exchange during the editing are included in the track changes version. The protocol should include all relevant documentation (ICF, study instruments, peer review, etc.) even if already submitted.

Please note that comments in the introductory paragraph are meant for the WHO Responsible Staff Member, though you may decide to share them with the PI.

PLEASE RESPOND TO THIS REVIEW SUMMARY WITHIN A three MONTH PERIOD, OR PROVIDE THE ERC SECRETARIAT A VALID JUSTIFICATION FOR THE DELAY.

The ERC considered this an important study. However, the committee had difficulties in assessing its social value as there seems to be no measurement of how effective the intervention is in changing attitudes and believes of health workers which would in terms provide evidence that the training has the desired impact. The ERC suggests to re-think the consent process so that data on ongoing illegal practices (carried out by health workers or other community members) can be gathered. Please see specific comments below.

A. Amendments (Response and change required)

This section includes queries and comments on your protocol, study instruments or the informed consent form for which the ERC requires your response and where relevant, appropriate amendments to the protocol, study instruments or the informed consent.

1. Protocol
   1.1. Please provide an amended proposal specifying the version number and/or date on each page.

In relation to the social value of the study:

1.2. The protocol states "The two principal aims of this research are to change attitudes and behavior of health care providers using systems based and social norms change approaches…". However, the study is not designed to measure the impact of the intervention which makes difficult to assess its social value.

The Responsible Officer (RO) kindly clarified, during the ERC meeting, that data that would allow to assess the impact of the intervention was not being collected because of its sensitivity -FGM is illegal in the countries where the study will take place. The ERC considered that in order not to comprise the study, data could be collected anonymously. Given that the study will randomize health centres to the intervention and control arms, an information session could take place at health facility level to explain the study to health personnel and information sheets explaining the study could be distributed. However, consent would not be required (please refer to CIOMS, Guideline 21).

1.3. In relation to the study objectives, the following comments may seems scientific issues but they have an ethical bearing as the study should be accurate in terms of what is able to achieve without creating expectations that cannot be fulfilled.

1.3.1. One of the primary outcomes of the study is "To determine if a health systems package that uses a social norm change approach improves health systems preparedness to provide FGM prevention and care within the antenatal care context".

1.3.1.1. Please clarify the care that is currently being provided at the study sites.

1.3.1.2. Please explain how this outcome will be evaluated since none of the study instruments submitted address care.

1.3.2. One of the secondary objectives (process evaluation) is "To understand how and why a behavioral intervention that uses a social norm change approach achieved its implementation outcomes, including the feasibility, acceptability, fidelity and coverage of the intervention". Please better define how each one of the outcomes will be achieved.

In relation to potential risks and benefits:

1.4. Anonymising the information would allow the study team to collect data on attitudes and behaviour and understand the percentage of health providers do/do not conduct female genital mutilations (FGM) and their reasons. It seems that without this information it is not possible to assess whether the intervention had an impact. Please address.

1.5. The protocol states that "…most ANC sites have only one provider per site" and that "The cluster unit size varies between on to three midwives/nurses at each site".

1.5.1. Please further clarify the process that will be followed to select participants for the training and the IDIs.

1.5.2. In case all health workers at the ANC site take part in the training, please specify how it will be ensured that routine services are not highly affected.

1.6. In relation to the questionnaire (page 56), Question 6 makes reference to a health talk on FGM in the waiting room. This activity is not described neither in the protocol nor in the consent document. Please address.

1.7. The exit questionnaire proposed for use with antenatal care clients -which is not validated, has in its majority sensitive questions that may be perceived as invasive given that they will be quickly asked to clients when leaving the facility. Please discuss alternative ways of collecting this information from patients.

1.8. Please specify the data analysis that will be carried out on the data being gathered.

2. Informed Consent Forms

2.1. Please simplify the consent forms. The wording used is difficult to follow.

B. Clarifications (Response required but change may not be required)

This section includes queries on your protocol, study instruments or the informed consent form for which the ERC requires to make changes to your protocol.

1. Protocol

1.1. In relation to the study timeline (6 months):

1.1.1. It seems ambitious, given the complex outcomes of the study, to consider that a 6 months period will suffice to conduct all study activities. Please address.

1.1.2. Please kindly clarify when the different activities will take place and the time lapse between them. For example, it is unclear at what point the intervention arm will receive the PCC training (e.g. immediately after the baseline survey, 3 months after the baseline survey?).

1.1.3. Please discuss whether three months (period between intervention and a second follow up data collection) is a reasonable time lapse to be able to detect a change in attitude and behaviour among health providers.

1.2. "Research capacity strengthening opportunities will be identified and developed to ensure that country research teams are fully engaged and that skills are developed to carry out independent analyses". Please clarify the needs identified, the skills that will be strengthened, and how this will be done within the study timelines.

1.3. Only primary health centers were selected for inclusion because the first contact is with the nurse. Please state the relative size of primary health centers in comparison with hospitals and the private sector.

1.4. It is unclear whether research members, data collectors or another member of the study team will conduct the interviews. Please clarify and specify the training and background of the interviewer.

1.5. "During month 6 of the study timeline, in-depth interviews with ANC providers from performing and non-performing sites". Please clarify what is meant by "performing/non-performing sites" and the criteria that will be use to categorize them.

1.6. Even though the Conceptual Framework "...recognizes the social norms aspects influencing health care providers and communities", women are referred to in the protocol as those who "...tend to be the decision makers in the household regarding FGM". Please clarify the statement or modify as appropriate.

C. Suggestions

This section consists of suggestions for alternative scientific or technical approaches or methods for conducting the research but which do not raise critical, ethical issues. These are meant to be helpful to investigators and are presented as suggestions for you to consider incorporating into a revised protocol. No response from you is required for any comment in this section. If, however, you do make changes to the protocol as a result of these suggestions, please submit the revised protocol to the ERC.

Researchers are suggested to number sections and subsections of the protocol to facilitate understanding of the different steps of the study.

Protocol ID: A65993 **Meeting Date**: 13/06/2019

Based on the above comments, the Committee has the following recommendation(s) for this proposal:

[ ] The proposal is ***Approved as submitted.*** No modifications are required.

[X] The proposal is ***Conditionally Approved; requires amendments and/or clarifications.*** Final approval is contingent upon an adequate response by the Principal Investigator, to the satisfaction of the reviewers or the Chair on behalf of the ERC.

[ ] The proposal is ***Not approved; requires additional information and/or rewriting.*** A revised version of the proposal should be re-submitted by the WHO responsible staff member as a new submission to the ERC for re-review by Committee.

[ ] The proposal is ***Rejected***. The proposal is ethically unacceptable, for the reasons stated above. The Principal Investigator may submit a new proposal that takes into consideration the ethical issues raised by the Committee. If you do not agree with the Committee's assessment, please feel free to submit an appeal to the Chair of the ERC, through the Secretariat.

NOTE: Final Approval of the Proposal is contingent upon submission of the following:

[ ] Local ethics approval(s) [ ] Other relevant documents

The ERC would like to receive a copy of the recommendations of the local ethics committee when available.

IMPORTANT

1. *Any changes to the proposal or to the attachments (informed consent/study instruments etc.) should be approved by ERC before being implemented.*
2. *The approval for this proposal is valid for a period of* ***one year only.***
3. *Please resubmit this proposal for a Continuing Review at least 2 months before the next re-approval period.*

Chairperson .... **Date**: 28.06.2019

Name: Peter Olumese

**FINAL APPROVAL**	**FOR THE SECRETARIAT**
Amendments and Clarifications to the proposal have been reviewed.<br>The protocol (Version: 6 Date: 25.04.19) and informed consent Forms (Dated: 25.04.19 V6) submitted on 13.08.2019 are approved by the ERC<br><br>**Chairperson** ....<br>PO/AL<br>**Name** ....<br>**Date** 09.09.2019	Amendments and Clarifications to be reviewed:<br>[ ] Electronically by ERC<br>[ ] by Primary reviewers<br>[ ] by Secretariat<br><br>Amendments approved / Clarifications accepted on<br>Local ERC approval(s) obtained on NA<br>Relevant Documents submitted on NA<br>Comments: NA<br><br>Signature Date 09/09/2019
